# Analysis of ATP7A Expression and Ceruloplasmin Levels as Biomarkers in Patients Undergoing Neoadjuvant Chemotherapy for Advanced High-Grade Serous Ovarian Carcinoma

**DOI:** 10.3390/ijms251810195

**Published:** 2024-09-23

**Authors:** David Lukanović, Sara Polajžer, Miha Matjašič, Borut Kobal, Katarina Černe

**Affiliations:** 1 Department of Gynecology, Division of Gynecology and Obstetrics, University Medical Centre Ljubljana, Zaloška 2, SI-1000 Ljubljana, Slovenia; david.lukanovic@kclj.si (D.L.); borut.kobal@kclj.si (B.K.); 2 Department of Gynecology and Obstetrics, Faculty of Medicine, University of Ljubljana, Šlajmerjeva 3, SI-1000 Ljubljana, Slovenia; 3 Institute of Pharmacology and Experimental Toxicology, Faculty of Medicine, University of Ljubljana, Korytkova 2, SI-1000 Ljubljana, Slovenia; sara.polajzer@mf.uni-lj.si; 4 Department of Education Studies, Faculty of Education, University of Ljubljana, SI-1000 Ljubljana, Slovenia; miha.matjasic@pef.uni-lj.si

**Keywords:** ATP7A, transporter, ceruloplasmin, ovarian cancer, high-grade serous carcinoma, chemoresistance, predictive marker

## Abstract

Ovarian cancer (OC), particularly high-grade serous carcinoma (HGSC), is a leading cause of gynecological cancer mortality due to late diagnosis and chemoresistance. While studies on OC cell lines have shown that overexpression of the ATP7A membrane transporter correlates with resistance to platinum-based drugs (PtBMs) and cross-resistance to copper (Cu), clinical evidence is lacking. The functionality of ceruloplasmin (CP), the main Cu-transporting protein in the blood, is dependent on, among other things, ATP7A activity. This study investigated ATP7A expression and CP levels as potential biomarkers for predicting responses to PtBMs. We included 28 HGSC patients who underwent neoadjuvant chemotherapy (NACT). ATP7A expression in ovarian and peritoneal tissues before NACT and in peritoneal and omental tissues after NACT was analyzed via qPCR, and CP levels in ascites and plasma were measured via ELISA before and after NACT. In total, 54% of patients exhibited ATP7A expression in pretreatment tissue (ovary and/or peritoneum), while 43% of patients exhibited ATP7A expression in tissue after treatment (peritoneum and/or omentum). A significant association was found between higher ATP7A expression in the peritoneum before NACT and an unfavorable CA-125 elimination rate constant k (KELIM) score. Patients with omental ATP7A expression had significantly higher plasma mean CP levels before NACT. Plasma CP levels decreased significantly after NACT, and higher CP levels after NACT were associated with a shorter platinum-free interval (PFI). These findings suggest that the ATP7A transporter and CP have the potential to serve as predictive markers of chemoresistance, but further research is needed to validate their clinical utility.

## 1. Introduction

Ovarian cancer (OC) is the second most frequent type of gynecologic cancer, and it has the highest mortality rate. The high mortality rate is the consequence of the clinical picture and diagnosis; most women already have symptoms connected with an advanced stage, which often spreads beyond the ovaries and involves metastases in the peritoneal cavity [1,2,3,4]. Consequently, a diagnosis is made at an advanced stage, when treatment is less effective (stages III and IV according to the International Federation of Gynecology and Obstetrics [FIGO]) [5]. The 5-year survival rate for women who are diagnosed with OC in the early stage is 93%, while the rate for those diagnosed in the advanced stage is 29% [6]. High-grade serous carcinoma (HGSC) is the most common malignancy of the ovary, with the likeliest origin in the epithelium of the fallopian tube fimbriae [7,8,9]. Primary cytoreductive/debulking surgery (PDS) to remove all visible tumor tissue is the standard primary treatment, followed by chemotherapy—adjuvant chemotherapy (ACT). Interval debulking surgery (IDS) following neoadjuvant chemotherapy (NACT), commonly comprising three/four cycles of chemotherapy, is an alternative treatment option for patients who are unable to undergo primary complete resection. Survival depends on the degree of optimal cytoreduction achieved [7,10]. Platinum (Pt)-based medicines (PtBMs) have been considered fundamental chemotherapies for the past four decades. Moreover, despite novel targeted drugs, carboplatin with added paclitaxel remains the standard first-line treatment for advanced OC [4]. Between 20 and 40% of patients do not respond to the primary therapy described. In addition, chemoresistant recurrence gradually develops in up to 80% of patients who initially respond well to treatment [11]. Pt-resistant OC is invariably a fatal disease; therefore, Pt sensitivity continues to be the major prognostic determinant [3,12,13]. Although resistance to PtBMs is considered multifactorial, reduced cellular Pt content is an important hallmark of resistance in Pt-resistant ovarian cell lines [3,14,15]. There are currently no validated molecular predictive biomarkers for Pt resistance or resistance to targeted therapies or prognostic biomarkers in clinical practice that could help inform treatment decisions and enable more personalized treatment [10,11]. Membrane transporters may play an important role in developing chemoresistance and influencing the prognosis of OC patients, but the precise contribution of these transporters has not been elucidated yet [16]. Copper-transporting ATPases—ATP7A and ATP7B—are membrane transporters that maintain cellular copper (Cu) homeostasis. To date, only Samimi et al. [17] have quantified ATP7A expression in tumor samples from OC patients before and after PtBM therapy, though the specific type of tissue sample was not specified. In that study, patients with increased post-treatment ATP7A expression (33.3% of patients) had worse actuarial survival, but there was no significant association between pretreatment and post-treatment ATP7A expression. However, the OC histology was variable, and detailed analyses were not performed on the most common histology—HGSC. Nakayama et al. [18] retrospectively investigated ATP7B expression in primary OC and its association with the chemotherapeutic response, finding that patients with ATP7B-positive carcinomas (30% of patients) had worse overall survival than those with ATP7B-negative tumors. However, this study provided limited information on the detailed histological subtypes of OC in relation to ATP7B expression. On the other hand, Elsnerova et al. [19] reported that ATP7A expression in tumor samples did not correlate with progression-free survival [19]. However, none of the published studies have evaluated ATP7A expression in NACT. Because this literature review has revealed a significant gap in understanding the role of ATP7A in OC and conflicting results in published studies, our research group has focused on further elucidating the potential of ATP7A as a biomarker in OC [3,4].

Ceruloplasmin (CP), the main Cu-transporting protein in the blood, is synthesized in the cell, and ATP7A is responsible for delivering Cu to CP. When Cu binds to CP, it can cross the plasma membrane via the ATP7A transporter [20,21]. Based on this relationship, we hypothesized that measuring CP levels in bodily fluids could be an indirect measure of ATP7A expression. Moreover, Huang et al. [22] previously explored CP levels in ascites as a potential prognostic biomarker for chemotherapy response in epithelial OC.

Our study aimed to investigate ATP7A expression and CP levels as potential biomarkers for predicting responses to PtBMs in patients with HGSC undergoing NACT. We analyzed ATP7A expression in various tissues, including the ovary before chemotherapy, the omentum after chemotherapy, and the peritoneum before and after chemotherapy with PtBMs. Additionally, we assessed CP levels in plasma and ascites before chemotherapy, as well as in plasma after chemotherapy. We also investigated the association between ATP7A expression and CP levels with chemotherapy response, measured by the CA-125 elimination rate constant K (KELIM) score and the platinum-free interval (PFI). Furthermore, we aimed to evaluate whether CP concentrations in ascites or plasma could indirectly indicate ATP7A transporter activity in HGSC patients.

## 2. Results

The study group comprised 28 Caucasian patients (all from Slovenia) with HGSC who were referred for NACT. Five of these patients did not complete the treatment regime or sample collection—primary surgery + NACT + IDS—owing to chemotherapy refusal, a change of treatment facility, death, or a lack of follow-up after the start of treatment. At the time of analyses, nine patients out of 28 had died, and 13 patients did not have detectable disease progression. The median follow-up time for survivors was 23.5 months (minimum of 13.1 months and maximum of 37.3 months). The 1-year cumulative survival was 82.1%. The summarized patient data are presented in Table 1.

International Federation of Gynaecology and Obstetrics (FIGO); neoadjuvant chemotherapy (NACT); C-reactive protein (CRP); Modeled CA-125 elimination constant K (KELIM); primary surgery (PS); interval debulking surgery (IDS); platinum-free interval (PFI).

### 2.1. ATP7A Expression in Different Tissues at Different Time Points

We analyzed CP levels in various fluids and ATP7A expression in different tissues at distinct time points before (at PS) and after 3–6 cycles of chemotherapy with PtBMs (at IDS) (Table 2).

**Figure 1:**
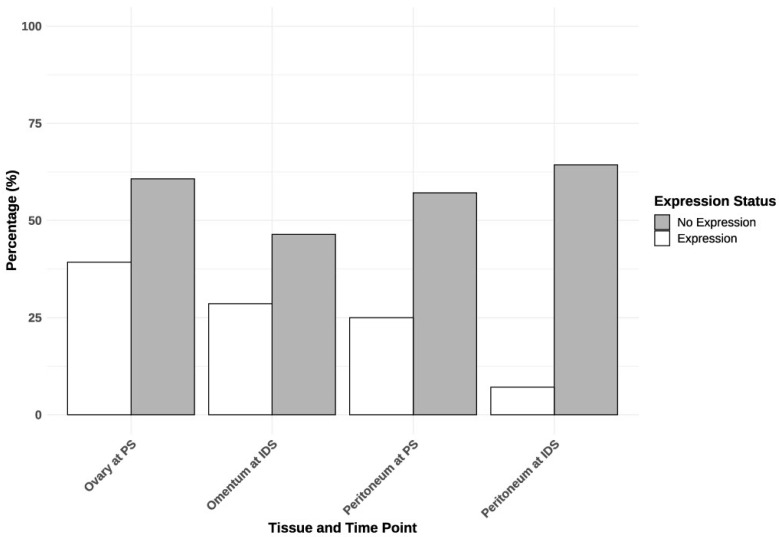
Expression of ATP7A in different tissues at different time points. PS—primary surgery; IDS—interval debulking surgery.

**Figure 2:**
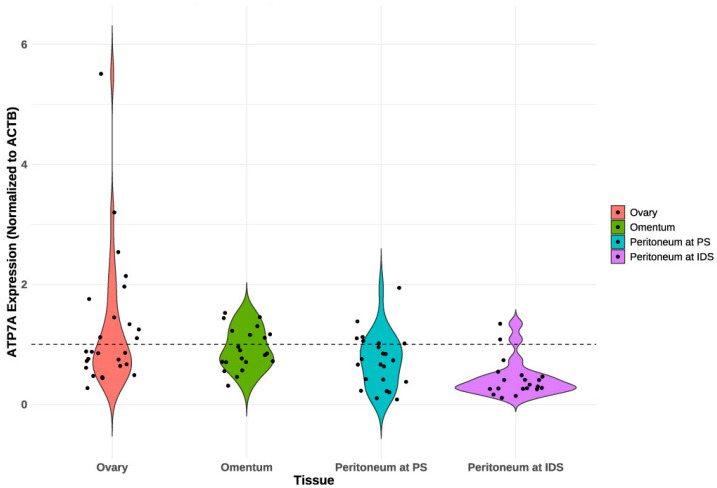
Violin plot of PCR-based ATP7A expression, normalized to the ACTB gene, across different tissues. ATP7A expression is categorized as positive (≥1, dashed line) and negative (<1) normalized to the reference gene. PS—primary surgery; IDS—interval debulking surgery.

At PS, 15 patients (54%) exhibited ATP7A expression in tissues (ovary or peritoneum), while only three patients (11%) demonstrated this expression in both tissues. At IDS, nine patients (43%) exhibited ATP7A expression in tissue (omentum or peritoneum), whereas only one patient (5%) demonstrated this expression in both tissues. Eleven patients (40.74%) had ATP7A expression in ovarian tissue at PS (Table 2 and Figure 1). Table 2 shows a decrease in ATP7A expression across different tissues following chemotherapy, particularly in the peritoneum, where expression drops significantly from 25% at primary surgery (PS) to just 7.1% at IDS. Figure 1 visually supports these findings by illustrating the distribution of ATP7A expression and non-expression in each tissue at different time points. Moreover, in Figure 2, we present a violin plot that displays the distribution of ATP7A expression across different tissues at different time points. The violin plot indicates considerable variation in ATP7A expression across different tissues, with the ovary exhibiting the highest and most variable expression levels.

We continued by analyzing the association between ATP7A expression in different tissues (Table 3). However, the analysis suggests a moderate association between ATP7A expression in the ovary at PS and the peritoneum at IDS (Cramér’s V = 0.408), but this association is not statistically significant (*p* = 0.068). Additionally, the patient’s age did not significantly correlate with ATP7A expression in different tissues (*p* = 0.147). No significant association was found between tumor origin (ovary, fallopian tube, or peritoneum) and ATP7A expression in the ovary (*p* = 0.578) or peritoneum at PS (*p* = 0.693). The distribution of ATP7A expression in the ovary appeared to be relatively uniform across different diagnostic sources, with no systematic association between these variables. Among patients with an ovarian origin, the majority (75%) exhibited no ATP7A expression, whereas 25% did demonstrate expression. In patients with a fallopian tube origin, the distribution was more uniform, with 58% exhibiting no expression and 42% demonstrating expression. In patients with a peritoneal origin, the distribution was evenly split, with 50% exhibiting no expression and 50% exhibiting expression.

### 2.2. Ceruloplasmin Levels in Different Fluids at Different Time Points

**Figure 3:**
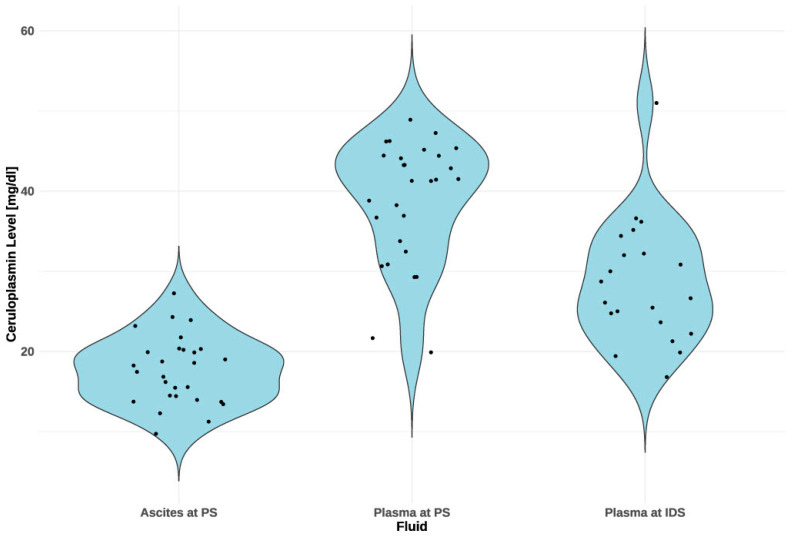
CP concentration levels in paired ascites and plasma samples before chemotherapy and in plasma samples after chemotherapy. CP levels in ascites are consistent and generally lower, centered around 20–25 mg/dL. In plasma at PS, CP levels are more variable, with a mean of 38.68 mg/dL. After chemotherapy (at IDS), the distribution shifts downwards to a mean of 28.21 mg/dL, but variability among patients remains. PS—primary surgery; IDS—interval debulking surgery.

**Figure 4:**
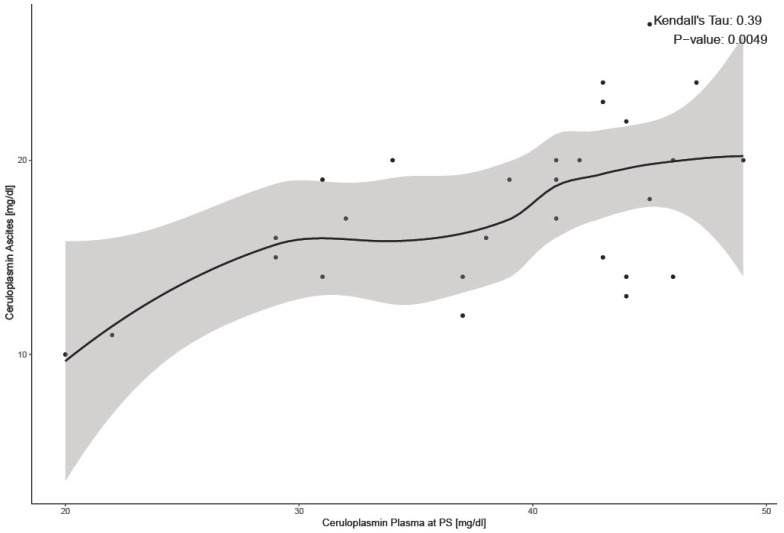
Correlation between CP in plasma at PS and ascites at PS. The shaded area in the plot represents the 95% confidence interval around the LOESS smoothing curve. PS—Primary surgery.

We continued our analyses with CP levels. The violin plots in the diagram (Figure 3) represent the distribution of CP levels across different fluids: ascites, plasma at PS, and plasma at IDS. Mean CP levels in plasma before chemotherapy (at PS) were 38.68 mg/dL ± 7.54 and, after 3–6 cycles of chemotherapy (at IDS), 28.21 mg/dL ± 8.00 (Table 2). CP levels in plasma at IDS were significantly lower than levels in plasma at PS (*p* = 0.001). Mean CP levels in ascites, 17.61 mg/dL ± 4.21, were significantly lower than plasma at PS, 38.68 mg/dL ± 7.54 (*p* < 0.001) There was a significant correlation between CP levels in ascites and plasma at PS (*p* < 0.001) (Figure 4).

Furthermore, we observed CP levels according to tumor origins. The dataset indicates that CP levels fluctuate according to the source of diagnosis (ovary, fallopian tube, or peritoneum); however, the observed fluctuations were not statistically significant (*p* = 0.057 for CP in plasma at PS; *p* = 0.428 for CP in ascites; *p* = 0.693 for CP in plasma at IDS). At PS, patients with a peritoneal source exhibited the highest ceruloplasmin levels (43.25 mg/dL ± 3.69). This trend persisted at IDS, with peritoneal sources still demonstrating the highest levels but with increased variability (32.20 mg/dL ± 11.19). CP levels in ascites are relatively consistent across the spectrum of diagnostic sources, with minor fluctuations.

Finally, we analyzed the correlations between CP levels and patient age. These correlations were very weak in all cases and not statistically significant (*p* = 0.953 for CP in plasma at PS; *p* = 0.146 for CP in ascites; *p* = 0.468 for CP in plasma at IDS). This suggests that age is not a strong determinant of CP levels.

To further explore the factors influencing CP levels, we conducted a regression analysis with C-reactive protein (CRP) as a predictor variable. CRP is a statistically significant predictor of CP at PS (*p* = 0.030), but it explains 14% of the variability. Although the model is statistically significant, the relatively low R^2^ value suggests that there are probably other important factors influencing CP that are not included.

### 2.3. Relationship between ATP7A Expression in Different Tissues and Ceruloplasmin Levels in Different Fluids

To analyze the association between ATP7A expression and CP levels, the CP levels were divided into two categories: low levels and high levels. The cut-off point was the median value, with levels below the median value classified as low and levels above the median value classified as high. Patients without ATP7A expression generally had higher CP levels in plasma (52.9%) at PS than those with ATP7A expression (54.5%), in both ovarian and peritoneal tissues. Regardless of whether ATP7A was expressed, CP levels at PS were evenly distributed between the low and high values (based on the median). Associations between ATP7A expression in different tissues and CP levels are presented in Table 4. There is a difference in the distribution of CP levels in the ascites between patients with ATP7A expression in the peritoneum at PS and those without expression. Most patients without ATP7A expression had high CP levels in the ascites (56.3%), whereas most patients with ATP7A expression had low CP levels in the ascites (71.4%). ATP7A expression in the omentum at IDS and CP levels in plasma at PS were significantly associated with a moderate effect size (*p* = 0.032). Moreover, patients with ATP7A expression in the omentum had higher mean CP levels in plasma at PS (42.63 mg/dL ± 5.97) than patients without expression (34.69 mg/dL ± 7.69), suggesting an association between ATP7A expression in the omentum and higher CP levels in plasma at PS (*p* = 0.022). Patients with ATP7A expression in the ovaries had higher mean CP levels in plasma at IDS (30.89 mg/dL ± 10.48) than those without expression (26.56 mg/dL ± 5.89). Moreover, the decrease in CP levels between PS and IDS was smaller in patients with ATP7A expression in the ovaries (5.70 mg/dL ± 10.56) than in the group without ATP7A expression (12.45 mg/dL ± 10.11); however, this difference was not statistically significant (*p* = 0.076). Later, we divided CP levels in plasma at IDS into quartiles. The first quartile (Q1) included values less than or equal to 22.925 mg/dL. The median, or second quartile (Q2), comprised values greater than those in Q1 but less than or equal to 26.1 mg/dL. The third quartile (Q3) includes values greater than those in Q2 but less than or equal to 31.425 mg/dl. Values that exceed 31.425 mg/dL were grouped into a fourth category. In Q1, Q2, and Q3, there was a higher proportion of patients without ATP7A expression in the ovary (60–75%), while in the group above Q3, there was a higher proportion of patients with ATP7A expression (57.1%). It seems that with increasing plasma CP concentrations at IDS (31.425 mg/dL > Q3), the proportion of patients with ATP7A expression increased, while at lower concentrations (Q1, Q2, and Q3), there was a higher proportion without ATP7A expression (*p* = 0.768). There was no difference between CP levels at PS and IDS in patients with ATP7A expression in the peritoneum at PS.

On the other hand, when looking at ATP7A expression in the peritoneum and omentum at IDS, patients with expression had a higher drop in plasma CP levels between PS and IDS than the group without expression; however, again, this was not statistically significant (*p* = 0.322, *p* = 0.149).

### 2.4. ATP7A Expression in Different Tissues and Responses to Chemotherapy

To assess tumor responses to chemotherapy, we analyzed the correlation between the PFI and KELIM. Ten patients had a recurrence less than 12 months after completing chemotherapy (PFI < 12 months). On the other hand, 14 patients had an unfavorable KELIM (Table 1). KELIM and PFI are positively correlated and statistically significant, suggesting that a lower KELIM score is associated with a shorter time to recurrence (*p* = 0.036). Furthermore, we analyzed the association between ATP7A expression and KELIM and PFI. There was a statistically significant association between ATP7A expression in the peritoneum at PS and the KELIM score (*p* = 0.024) (Table 5). Patients with ATP7A expression in the peritoneum at PS had a higher proportion of unfavorable outcomes (100%) than patients without ATP7A expression, who had a higher proportion of favorable outcomes (53.8%). There was no significant association between the ATP7A expression in different tissues at different time points and PFIs (Table 5). However, the association between ATP7A expression in the peritoneum at IDS and the PFI approaches statistical significance (*p* = 0.068), indicating a moderate relationship. This suggests that ATP7A expression in the peritoneum at IDS may have a closer association with the PFI.

### 2.5. Ceruloplasmin Levels in Plasma and Responses to Chemotherapy

Our final objective was to determine the association between CP levels and tumor responses to chemotherapy. We analyzed CP levels compared with the PFI and KELIM. Patients with a PFI of <12 months had significantly higher CP levels at IDS than did patients with a PFI of >12 months (*p* = 0.036). The difference between CP in plasma at PS and IDS was lower in the group with a PFI of <12 months (6.96 mg/dL) than the group with a PFI of >12 months (11.16 mg/dL), but this was not statistically significant (*p* = 0.391). Moreover, the difference between plasma CP at PS and IDS was also lower in the group with an unfavorable KELIM (8.61 mg/dL) than the group with a favorable KELIM (11.45 mg/dL), but this was not statistically significant (*p* = 0.576).

## 3. Discussion

The mechanisms of chemoresistance have been thoroughly studied in many cancers. In the last decade, there have not been any significant breakthroughs in preventing or treating chemoresistant cancers, particularly in OC [23,24,25]. There are currently no validated biomarkers that predict chemoresistance in OC or could provide a more accurate and prognostically relevant subclassification of HGSC that might predict survival or responses to chemotherapy. Although many studies have proven the role of intrinsic and acquired chemoresistance in OC, the therapeutic capacities remain limited [4,26]. As highly polar molecules, it is very difficult for Pt compounds to freely cross the cell membrane, but they can be transported by Cu-transporting proteins. The coordination chemistry of platinum (Pt2+) is very similar to that of copper (Cu1+), which is why it can bind to its transporters and other proteins that mediate Cu homeostasis [3]. In vitro studies [27,28,29] on OC cell lines have shown that ATP7A, a crucial Cu membrane transporter, plays an important role in chemoresistance; however, clinical studies investigating ATP7A’s potential to predict responses to therapy and prognosis are still lacking. ATP7A is primarily studied in related disorders such as Menkes disease and occipital horn syndrome, and its role in copper transport is well-documented. Pathogenic variants in ATP7A lead to these conditions, with recent findings by De Feyter et al. [30] indicating that CP is a more sensitive biomarker for these disorders than serum Cu. Additionally, Liu et al. [31] discovered that decreased ATP7A mRNA expression and elevated CP levels are associated with oxidative stress and disturbances in neuronal metabolism, contributing to the pathophysiology of major depressive disorder (MDD).

While previous studies by Samimi et al. [17] and Elsnerova et al. [19] have explored ATP7A expression in OC, they have notable limitations, particularly in their failure to adequately address tumor heterogeneity, a key feature of OC. Although HGSC is the dominant OC subtype seen in the clinic, most preclinical studies that have investigated Pt resistance have been performed on non-serous cell lines, and therefore may not apply to serous-type-specific therapy. Moreover, increasing studies are showing differences between and within individual OC types and subtypes [10]. The probability of a Pt response in low-grade serous, clear-cell, and mucinous OC is poor. The response to first-line chemotherapy in HGSC is better, but relapse and eventual treatment failure are common [10]. In addition, studies have demonstrated tumor heterogeneity at the individual patient level, making the cure even more difficult [4].

Our study is the first to analyze ATP7A expression in samples from patients with HGSC who underwent NACT, allowing us to collect different tissues and fluid samples before chemotherapy and at IDS. Our previous findings reaffirmed the non-superiority of NACT + IDS compared with PDS for treating epithelial OC and building on this, we carefully selected patients likely to benefit from NACT + IDS, avoiding unfavorable procedures [32,33]. Although Samimi et al. [17] were the first to analyze ATP7A expression before and after chemotherapy, our study is the only one that focuses on patients with HGSC. We specifically and separately examined ATP7A expression in ovarian tissue, the peritoneum, and the omentum, making our study the first to provide a detailed tissue-specific analysis in a patient population. The ovaries are the primary tissues for histopathological evaluation, and they showed the highest percentage of ATP7A expression among patients at PS, with 11 exhibiting expression. This expression was also the highest and most variable when normalized to the endogenous control, ACTB (Figure 2). Altogether, 54% of patients had ATP7A expression in tissues at PS, and after 3–6 cycles of chemotherapy with PtBMs, the percentage of patients with ATP7A expression dropped to 43%. This drop was highest in the peritoneum, as this is the only tissue we evaluated before and after chemotherapy. ATP7A expression in the peritoneum was more variable in PS, whereas the expression levels became lower in IDS, reflecting the potential impact of chemotherapy in reducing ATP7A expression in the peritoneum. Although the association between expression in different tissues at different times has not been statistically proven, our findings reveal a significant association between ATP7A expression in the peritoneum at PS and an unfavorable KELIM score. A higher KELIM score is associated with a better response to chemotherapy and improved survival outcomes [34,35]. The observed association suggests that ATP7A expression in the peritoneum at PS could be a marker for poor chemotherapy response, potentially because of its role in mediating Pt resistance. This finding is particularly relevant given the heterogeneity of HGSC, where different tissue sites within the same patient can exhibit varying chemoresistance levels. There was a statistically significant correlation between the KELIM score and the PFI in our cohort. We should also not dismiss the trend towards an association between ATP7A expression in the peritoneum at PS and IDS and PFIs, but this did not reach statistical significance in our study. The PFI is a crucial clinical endpoint, reflecting the duration between the end of PtBM treatment and recurrence [36].

In addition to ATP7A, we examined CP levels as a potential biomarker for chemoresistance. CP is the primary Cu-carrying protein in the blood and plays a key role in Cu homeostasis [37]. We statistically proved a correlation between CP levels in plasma and ascites, one that has not yet been mentioned in the literature. This significant correlation suggests a consistent relationship between CP concentrations in different biological fluids. Our results indicate that mean plasma CP levels at the time of PS were higher than those in ascites, supporting the hypothesis that CP may act not only as a marker of Cu metabolism but also as an indicator of systemic inflammation [38]. Our analyses of a positive correlation between CP levels and CRP at PS support this hypothesis. However, CRP explains only a small part of the variability in CP levels (14%). In our cohort, the mean plasma CP levels at PS ranged from 19.89 to 48.91 mg/dL, with an average of 38.68 mg/dL ± 7.54, slightly higher than the normal serum CP concentration range of 20 to 40 mg/dL [39]. Elevated serum CP levels have been linked to various cancers, including oral, thyroid, prostate, colon, and breast cancers, where they are associated with tumor invasion and metastasis [40,41,42]. On the other hand, serum CP levels are reduced in hepatic disorders, Wilson’s disease, aceruloplasminemia, and vitamin C overdose. It has been observed that altered CP levels follow chemotherapy or radiation in laryngeal, cervical, and breast cancers, with greater post-treatment decreases correlating with improved outcomes [22,43,44]. Notably, there are currently no data on the effects of these treatments on CP levels in OC. Moreover, Weijl et al. [45] demonstrated that CP levels decline following chemotherapy but typically revert to baseline levels before commencing the subsequent cycle. This agrees with our findings showing that CP levels decreased less in patients with shorter PFIs and suggests that those with earlier recurrence tend to have higher mean CP levels at IDS (after chemotherapy), indicating a potential role for CP as a marker of poor prognosis. Although Huang et al. [22] reported statistically significant higher CP levels in intrinsically chemoresistant ascites than chemosensitive ascites (with average concentrations of 19.2 mg/dL and 15.75 mg/dL, respectively), our study did not confirm this finding. However, the average CP concentrations of 19.2 mg/dL in our study are comparable to CP ascites levels in a study by Huang et al. [22]. Our analysis revealed a significant association between CP levels in plasma at IDS and PFI. Specifically, patients with lower CP levels following chemotherapy demonstrated a more favorable PFI. This suggests that reduced CP levels after chemotherapy may indicate an improved therapeutic response. While the role of ATP7A in Cu transport led us to hypothesize that CP levels indirectly reflect ATP7A activity, we found a statistically significant association between ATP7A expression in pretreatment omentum and plasma CP levels, as patients with ATP7A expression had higher plasma CP levels at PS than patients without ATP7A expression. Moreover, patients with ATP7A expression in the omentum also had higher CP levels in ascites at PS. No other statistical association was found. This may be due to the complex regulation of Cu homeostasis involving multiple proteins and pathways beyond ATP7A. There is a trend towards higher plasma CP levels after chemotherapy in patients with ATP7A expression in ovarian tissue than those without expression, but this is not statistically significant.

It is essential to consider the potential limitations of the present study to ensure the reliability of its findings. The primary limitation of this study is the relatively small sample size, which is largely attributable to the focus on patients with advanced HGSC undergoing NACT. The specific subset, along with the strict inclusion criteria for histologically confirmed HGSC, resulted in a limited number of eligible participants. Furthermore, the restricted sample size was influenced by the single-center study design, given that only three centers in Slovenia treat advanced ovarian cancer. Furthermore, the loss of patients to follow-up resulted in a reduction of the cohort size, which consequently affected the statistical power of this study. Although a control group was not included in this study, intra-patient comparisons enabled the assessment of biomarker changes over time, thereby providing valuable preliminary insights into ATP7A and CP as predictive markers of chemoresistance in HGSC patients. Secondly, it is important to consider that successful molecular analyses of human solid tissues require intact biological material with well-preserved nucleic acids, proteins, and other cell structures [46]. A potential limitation is that the pre-analytical handling of the material, which occurred in the operating theatre, was beyond the control of the laboratory personnel. This is one of the earliest critical steps in the process, with the potential to influence the quality of the sample. However, variability between tissue samples from primary tumours and metastatic sites is possible. Tumour heterogeneity, particularly in HGSC, is a critical factor influencing chemoresistance, progression, and metastatic potential. Both intra-tumoral heterogeneity (ITH) and inter-tumoral heterogeneity (variation between primary and metastatic sites) are well-documented in HGSC and are major contributors to the observed variability in treatment response and biomarker expression [47]. Metastatic lesions frequently display distinctive biological and molecular characteristics compared with the primary tumor owing to genetic divergence and disparate microenvironmental influences [48]. The literature supports the notion that HGSC tumors exhibit extensive ITH, driven by both clonal evolution and the presence of cancer stem cells. This heterogeneity not only contributes to variations in gene expression but also leads to distinct subclonal populations within the same tumor. As noted in a recent review by Roberts et al., ITH is closely associated with chemoresistance, metastasis, and recurrence, with different clonal populations responding differently to therapeutic intervention [49]. As there is no correlation between ATP7A expression in the ovaries and omentum, it can be surmised that ATP7A expression in primary tumors may not accurately reflect its expression in metastases, complicating the interpretation of the results. Furthermore, we recognize that p73 is important in regulating ATP7A and can affect resistance to platinum-based treatments. Although we did not collect p73 data in this study, it is known to play a role in cisplatin-induced apoptosis through calcium/calpain-dependent mechanisms. Future research should include p73 expression to better understand its connection to ATP7A and chemoresistance [50].

Choosing the correct treatment strategy is crucial for improving survival in patients with advanced OC; however, the criteria for selecting the most effective therapies remain unclear. Biomarkers that predict a patient’s risk of developing chemoresistance to PtBMs would enable more tailored treatments, potentially sparing patients from the severe side effects of ineffective therapies. Our recent work identified EpCAM-positive extracellular vesicles in the ascites of patients with advanced HGSC as potential prognostic biomarkers, capable of predicting early recurrence and suggesting a likelihood of more aggressive tumor biology and chemoresistance [11]. Further investigations of this aspect would facilitate a more comprehensive understanding of ATP7A’s role. These findings will contribute to the incremental progress being made in developing new strategies to overcome chemoresistance, a central challenge in the field of OC research. It is increasingly evident that targeting a single mechanism is insufficient for effective cancer treatment, underscoring the shift toward personalized therapy.

## 4. Materials and Methods

### 4.1. Study Design

Our study was a prospective clinical trial on patients with a diagnosis of advanced-stage (FIGO stage III or IV) primary HGSC of the ovaries, fallopian tubes, or peritoneum diagnosed between January 2021 and June 2023 at the Department for Gynecology, University Medical Centre Ljubljana. The clinical trial was registered at Clinical Trials with reference number NCT05490407. Before analysis, all patients received detailed oral and written information about the research and procedures, and they signed informed consent forms regarding the analysis of their samples for our research. The trial was approved by the National Medical Ethics Committee of the Republic of Slovenia on 26 June 2019 (Nr: 0120-316/2019/3).

We considered the following inclusion criteria: diagnosis of primary HGSC with FIGO stage III or IV independently from tumor grading; absence of concomitant malignant neoplasms; and patients referred for NACT. We excluded patients referred for adjuvant chemotherapy (ACT) and all patients with a nonepithelial histologic type.

**Figure 5:**
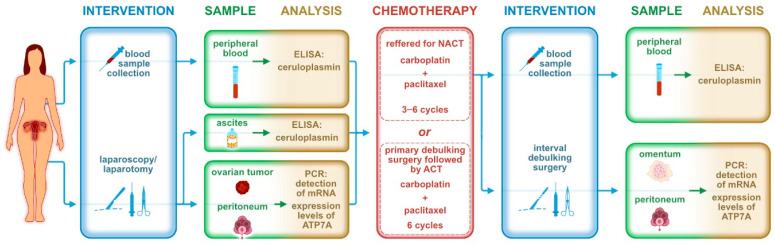
Chronological description of sample collection during the trial.

We applied 6 treatment courses of ACT; as a part of NACT, patients received 3–6 courses before IDS and 0–3 courses after surgical treatment. The standard ACT/NACT regimen for HGSC is based on carboplatin in combination with paclitaxel. When a patient’s general condition did not permit this combination, carboplatin was applied as a monotherapy. For maintenance therapy, PARP (poly ADP-ribose polymerase) inhibitors such as niraparib or olaparib were later added. Bevacizumab, an anti-vascular endothelial growth factor (anti-VEGF) antibody, was included in the chemotherapy regimen when the patient’s general condition permitted. For each patient, blood, ascites, and tissue samples were collected during the initial treatment and at PS, and additional blood and tissue samples were collected at IDS. Figure 5 is a chronological description of the sample collection.

### 4.2. Study Sample

**Figure 6:**
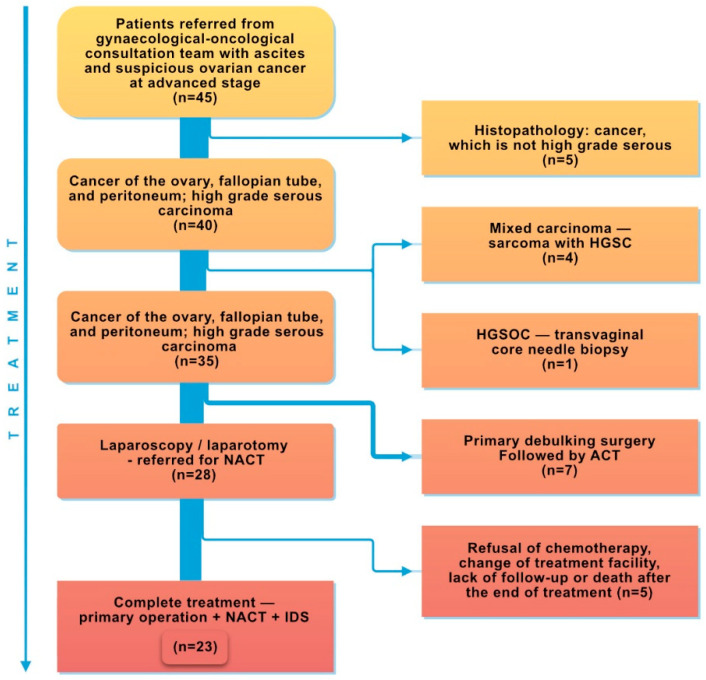
Patient selection flowchart.

Initially, the study included patients with suspected ovarian cancer (45 women) referred for laparoscopy/laparotomy depending on disease advancement, general health status, and local status determined based on gynecological examination and computed tomography. Following confirmation of the diagnosis of malignant ovarian, fallopian tube, or peritoneal cancer with the HGSC subtype via histopathological examination, the patients were qualified for further study stages. Five patients with non-HGSC cancer diagnosed during histopathological examination were excluded. The study continued with 40 patients with HGSC, who commenced chemotherapy. However, another 4 patients were rejected owing to mixed carcinoma (sarcoma with HGSC) and 1 patient did not undergo laparoscopy/laparotomy for the initial treatment. Figure 6 is a patient selection flowchart. Among the 35 patients, 7 received adjuvant therapy after PDS, and after subsequent laparoscopy or exploratory laparotomy, 28 were referred for NACT. Out of the 28 patients referred to NACT, 5 did not complete the treatment regime—primary surgery + NACT + IDS—owing to chemotherapy refusal, a change of treatment facility, death, or a lack of follow-up after the end of treatment.

### 4.3. Sample Collection and Storage

Venous blood samples for CP analysis were obtained while the patients were hospitalized for preoperative preparation before PS and IDS. In total, 6 mL of peripheral blood was collected in BD Vacutainer^®^ EDTA tubes (BD, Columbus, NV, USA) and left on ice for a maximum of 30 min before centrifuging the sample at 1000× *g* at 4 °C for 15 min. At the beginning of the operation, immediately after entry to the abdominal cavity, 50 mL of ascites was aspirated into a sterile syringe and immediately transferred into a conical tube, which was kept on ice until centrifugation at 1000× *g* for 15 min at 4 °C within for 15 min. Plasma and ascites supernatants were then stored in aliquots at −80 °C until analysis. In accordance with the standard treatment procedure, the primary tumor tissue removed during surgery was sent to histopathology for analysis. At least 1 cm3 of tissue was collected for ATP7A detection and then flash-frozen in liquid nitrogen and stored at −80 °C until analysis.

### 4.4. ELISA Analysis of Ceruloplasmin

CP concentrations were measured using a human ceruloplasmin (CP/CER) ELISA kit (CSBE08383h, CUSABIO, Wuhan, China). The analysis was performed according to the manufacturer’s protocol. The optical density was measured using a Synergy HT microplate reader (Bio Tek, Shoreline, WA, USA) at 450 nm. A second wavelength at 540 nm was used as a reference to subtract the background. A seven-point standard curve ranging from 0.156 to 10 ng/mL was generated using Curve Expert 1.4. We chose a curve with a 4-parameter fit. The lower limit of detection was less than 0.039 ng/mL. Each sample was tested in triplicate.

### 4.5. Isolation of RNA and qPCR of ATP7A

The High-Capacity cDNA Reverse Transcription kit (Thermo Fisher Scientific, Waltham, MA, USA) was used to create complementary cDNAs after RNA was extracted from tissue using the peqGold total RNA kit (VWR International GmbH, Vienna, Austria). Both RNA isolation and reverse transcription were performed according to the manufacturer’s guidelines. qPCR tests were performed to assess ATP7A expression. For the ACTB and ATP7A genes, we used pairs of oligonucleotides from ORIGENE (OriGene Technologies GmbH, Herford, Germany) (Table 6). To perform qPCR, we used a LightCycler 480 (Roche Diagnostics, Basel, Switzerland) and a HOT FIREPol EvaGreen qPCR Supermix, 5X (Solis, BioDyne, Tartu, Estonia) in accordance with the manufacturer’s guidelines. Cycling conditions were set at 95 °C for 12 min, followed by 40 cycles at 95 °C for 15 s and 60 °C for 30 s, along with a melting curve analysis. Before testing, each sample was diluted to a final concentration of 10 ng/µL. All samples were quantified in duplicate. A relative standard curve was created by preparing a dilution series of cDNAs, and the data were absolutely quantified using the second-derivative maximum method (LightCycler 480, software version 1.5; Roche Diagnostics, Basel, Switzerland). The internal housekeeping gene *beta-actin* (ACTB) was the standard for all data normalization. ATP7A expression in tissue samples was corrected with the ACTB housekeeping gene.

### 4.6. Parameters for Assessing Tumor Responses to Chemotherapy

The PFI is the time between the last dose of PtBM chemotherapy and disease recurrence, and it is one of the most important prognostic factors in cancer outcomes. Some works in the literature [12,50] categorize PFI into four subgroups: PFI > 12 months, PFI 6–12 months (partially platinum-sensitive disease), PFI < 6 months (platinum-resistant disease), and PFI < 1 month (refractory disease, defined as disease progression during systemic therapy). However, this concept has recently evolved to distinguish between two categories: the ‘platinum is an option’ category (group 1: PFI > 12 months, indicating platinum sensitivity) and the ‘platinum is not an option’ category (group 2: PFI < 12 months, indicating platinum resistance).

We applied the CA-125 elimination rate constant K (KELIM) score to assess tumor responses to chemotherapy. Using the online tool developed by You et al. [34,35] (https://www.biomarker-kinetics.org/CA-125-neo, accessed on 1 August 2024), we entered the date of every NACT cycle for each patient and the CA-125 levels within the first 100 days of NACT, including, at minimum, the values before cycles 2, 3, and 4, as described in the initial study [49]. The algorithm on the website yielded KELIM scores, predicted the probability of complete cytoreduction and platinum sensitivity recurrence, and projected overall survival rates. These values were entered as data points in our data collection. A higher value should be understood as a faster CA-125 elimination rate and, therefore, higher chemosensitivity. The KELIM value was dichotomized into a KELIM score: std KELIM < 1.0 was considered unfavorable and std KELIM ≥ 1.0 was considered favorable.

### 4.7. Statistical Analysis

All statistical analyses were performed using IBM SPSS Statistics, version 29 (IBM Corporation, Armonk, NY, USA). Continuous variables were described using the mean and the median with interquartile range (25–75%). Categorical variables were described using frequencies. To determine the association between ATP7A expression and clinical parameters, qPCR results were categorized as positive (≥1) or negative (<1), while average ATP7A levels normalized to the reference gene were used to show elevated/reduced expression. The normality of distribution was tested with the Kolmogorov–Smirnov and Shapiro–Wilk tests. The results indicated that most of these variables, including ceruloplasmin levels, were not approximately normally distributed, particularly when divided into groups. The parametric ANOVA and nonparametric Mann–Whitney tests compared the distribution of continuous variables among different patient groups. Kendall’s Tau-b correlation coefficient assessed correlations between continuous variables. Moreover, linear regression analysis explored the potential predictive relationship between CRP and CP levels, particularly in the PS context. This approach allowed us to determine whether CRP could significantly predict CP levels while accounting for other influencing factors. The regression model’s fit was evaluated using the adjusted R^2^ value, providing insight into how much of the variability in CP levels could be explained by CRP alone. Additionally, residual plots were examined to ensure that the assumptions of linear regression were met, such as homoscedasticity and normality of residuals. All statistical tests were two-sided, with the level of significance set to 0.05.

## 5. Conclusions

In conclusion, the present study investigates ATP7A expression and CP levels as potential biomarkers for predicting response to PtBMs in patients with HGSC. All patients underwent NACT, which enabled the collection of various tissue and fluid samples prior to the commencement of chemotherapy and at the IDS. Our findings indicate that ATP7A expression in the peritoneum prior to NACT is associated with an unfavorable KELIM score, which in turn is linked to a heightened risk of chemoresistance and poorer survival outcomes. Furthermore, a statistically significant correlation was observed between the KELIM score and PFI in the cohort under investigation. Despite the highest levels of ATP7A expression being detected in ovarian tissue, no correlation was found between this expression and the KELIM score. Following chemotherapy, CP plasma levels exhibited a statistically significant decline. Moreover, patients with shorter PFI demonstrated significantly elevated mean CP levels at the time of IDS. Accordingly, elevated CP plasma levels at IDS may indicate an increased risk of early recurrence and potentially more aggressive tumor biology. Furthermore, a significant correlation was identified between elevated CP concentrations in plasma prior to treatment and ATP7A expression in the omentum. Given that the omentum is a primary site of metastasis in HGSC, further investigation into the role of CP plasma levels before chemotherapy is warranted, despite the absence of an association between higher CP levels before chemotherapy and shorter PFI. These findings highlight the complex nature of chemoresistance and suggest that while ATP7A and CP may contribute to chemotherapy responses, further research with larger cohorts is needed to validate these biomarkers and fully understand their clinical implications.

## Figures and Tables

**Table 1:** Patient characteristics.

Variable	Category	n	%	Mean	SD
Age	Numerical variable	28	100	63.04	8.83
Menopause	Yes	25	89.30	-	-
	No	3	10.70	-	-
BMI	Numerical variable	28	100	28.23	6.03
Smoking	Yes	7	25.00	-	-
	No	21	75.00	-	-
Alcohol consumption	Yes *	4	14.00	-	-
	No	24	86.00	-	-
FIGO Stage	III C	17	60.70	-	-
	IV A	8	28.60	-	-
	IV B	3	10.70	-	-
Origin of the tumor	Ovary	8	28.60		
	Fallopian tube	12	42.90	-	-
	Peritoneum	8	28.60	-	-
Chemotherapy	Yes, NACT	25	89.30		
	Yes, palliative	1	3.56		
	No	2	7.14		
CRP at PS	Numerical variable	28	100	65.39	53.90
CRP at IDS	Numerical variable	23	82.0	10.39	18.39
KELIM Score	<1	14	60.90	-	-
	≥1	9	39.10	-	-
PFI	<12 months	10	35.70	-	-
	≥12 months	18	64.30	-	-

* more than 7 units per week or more than 1 unit per day and more than 3 units on one occasion.

**Table 2:** Levels of Ceruloplasmin at different time points and fluids and ATP7A expression in different tissues at different time points.

Variable	Category	n *	%	Mean [mg/dL]	SD [mg/dL]
mRNA expression of ATP7A in ovaries at PS	0	16	59.26	-	-
	1	11	40.74	-	-
mRNA expression of ATP7A in peritoneum at PS	0	16	57.10	-	-
	1	7	25.0	-	-
mRNA expression of ATP7A in peritoneum at IDS	0	18	64.30	-	-
	1	2	7.10	-	-
mRNA expression of ATP7A in omentum at IDS	0	13	46.40	-	-
	1	8	28.60	-	-
Ceruloplasmin in plasma at PS	Numerical variable	28	100.0	38.68	7.54
Ceruloplasmin in ascites at PS	Numerical variable	28	100.0	17.61	4.21
Ceruloplasmin in plasma at IDS	Numerical variable	21	75.0	28.21	8.00

* Due to limitations in the availability of tissue samples, it was not possible to obtain RNA from all patients. Primary surgery (PS); interval debulking surgery (IDS); ATP7A expression is categorized as positive (≥1) or negative (<1), normalized to the reference gene.

**Table 3:** Associations between ATP7A expression in different tissues.

ATP7A Expression between Different Tissues	Chi-Square Value	df	Cramér’s V	*p*-Value
Ovary at PS and Peritoneum at PS	0.058	1	0.051	0.809
Ovary at PS and Peritoneum at IDS	3.333	1	0.408	0.068
Ovary at PS and Omentum at IDS	0.151	1	0.085	0.697
Peritoneum at PS and Omentum at IDS	0.878	1	0.227	0.349
Peritoneum at PS and Peritoneum at IDS	2.15	1	0.112	0.643
Peritoneum at PS and Omentum at IDS	0.093	1	0.068	0.761

PS—primary surgery; IDS—interval debulking surgery.

**Table 4:** Association between ATP7A expression in different tissues and ceruloplasmin levels in different fluids.

ATP7A Expression and Ceruloplasmin Levels	Chi-Square Value	df	Cramér’s V	*p*-Value
Ovary at PS and Ceruloplasmin in Plasma at PS	0.150	1	0.073	0.699
Ovary at PS and Ceruloplasmin in Ascites at PS	1.348	1	0.219	0.246
Peritoneum at PS and Ceruloplasmin in Plasma at PS	0.100	1	0.066	0.752
Peritoneum at PS and Ceruloplasmin in ascites at PS	1.495	1	0.255	0.221
Peritoneum at IDS and Ceruloplasmin in Plasma at IDS	0.000	1	0.000	1.000
Omentum at IDS and Ceruloplasmin in Plasma at PS	5.639	1	0.510	0.032
Omentum at IDS and Ceruloplasmin in Ascites at PS	0.152	1	1.000	0.085
Omentum at IDS and Ceruloplasmin in Plasma at IDS	1.147	1	0.219	0.284

PS—primary surgery; IDS—interval debulking surgery.

**Table 5:** Association between ATP7A expression in different tissues and KELIM and PFI.

	Tissue and Variable Combination	Chi-Square Value	df	Cramér’s V	*p*-Value
KELIM	mRNA expression of ATP7A in ovaries at PS	0.209	1	0.095	0.648
	mRNA expression of ATP7A in peritoneum at PS	5.115	1	0.519	0.024
	mRNA expression of ATP7A in peritoneum at IDS	0.952	1	0.218	0.329
	mRNA expression of ATP7A in omentum at IDS	0.404	1	0.095	0.525
PFI	mRNA expression of ATP7A in ovaries at PS	0.749	1	0.164	0.387
	mRNA expression of ATP7A in peritoneum at PS	2.218	1	0.255	0.136
	mRNA expression of ATP7A in peritoneum at IDS	3.333	1	0.408	0.068
	mRNA expression of ATP7A in omentum at IDS	0.777	1	0.164	0.378

PS—primary surgery; IDS—interval debulking surgery.

**Table 6:** Primer sequences for ACTB and ATP7A genes.

Gene Symbol	Gene Name	Primer Sequence
ACTB	*Beta-Actin*	F: 5′-CACCATTGGCAATGAGCGGTTC-3′ R: 5′-AGGTCTTTGCGGATGTCCACGT-3′
ATP7A	*ATPase copper transporter alpha*	F: 5′-CCCTCTAGGAACAGCCATAACC-3′ R: 5′-ATACCACAGCCTGGCACAACCT-3′

## Data Availability

The original contributions presented in this study are included in this article; further inquiries can be directed to the corresponding author.

## References

[1] Ferlay J., Soerjomataram I., Dikshit R., Eser S., Mathers C., Rebelo M., Parkin D.M., Forman D., Bray F. (2015). Cancer incidence and mortality worldwide: Sources, methods and major patterns in GLOBOCAN 2012. Int. J. Cancer.

[2] Sant M., Chirlaque Lopez M.D., Agresti R., Sánchez Pérez M.J., Holleczek B., Bielska-Lasota M., Dimitrova N., Innos K., Katalinic A., Langseth H. (2015). Survival of women with cancers of breast and genital organs in Europe 1999–2007: Results of the EUROCARE-5 study. Eur. J. Cancer.

[3] Lukanović D., Herzog M., Kobal B., Černe K. (2020). The contribution of copper efflux transporters ATP7A and ATP7B to chemoresistance and personalized medicine in ovarian cancer. Biomed. Pharmacother..

[4] Lukanović D., Kobal B., Černe K. (2022). Ovarian Cancer: Treatment and Resistance to Pharmacotherapy. Reprod. Med..

[5] Labidi-Galy S.I., Papp E., Hallberg D., Niknafs N., Adleff V., Noe M., Bhattacharya R., Novak M., Jones S., Phallen J. (2017). High grade serous ovarian carcinomas originate in the fallopian tube. Nat. Commun..

[6] De Angelis R., Sant M., Coleman M.P., Francisci S., Baili P., Pierannunzio D., Trama A., Visser O., Brenner H., Ardanaz E. (2014). Cancer survival in Europe 1999–2007 by country and age: Results of EUROCARE-5—A population-based study. Lancet Oncol..

[7] National Comprehensive Cancer Network NCCN Clinical Practice Guidelines in Oncology (NCCN Guidelines^®^): Ovarian cancer (Version 1.2024). https://www.nccn.org/professionals/physician_gls/pdf/ovarian.pdf.

[8] Meinhold-Heerlein I., Fotopoulou C., Harter P., Kurzeder C., Mustea A., Wimberger P., Hauptmann S., Sehouli J. (2016). The new WHO classification of ovarian, fallopian tube, and primary peritoneal cancer and its clinical implications. Arch. Gynecol. Obstet..

[9] Lisio M.A., Fu L., Goyeneche A., Gao Z.H., Telleria C. (2019). High-Grade Serous Ovarian Cancer: Basic Sciences, Clinical and Therapeutic Standpoints. Int. J. Mol. Sci..

[10] Colombo N., Sessa C., du Bois A., Ledermann J., McCluggage W.G., McNeish I., Morice P., Pignata S., Ray-Coquard I., Vergote I. (2019). ESMO–ESGO consensus conference recommendations on ovarian cancer: Pathology and molecular biology, early and advanced stages, borderline tumours and recurrent disease. Ann. Oncol..

[11] Herzog M., Verdenik I., Kobal B., Černe K. (2024). Higher EpCAM-Positive Extracellular Vesicle Concentration in Ascites Is Associated with Shorter Progression-Free Survival of Patients with Advanced High-Grade Serous Carcinoma. Int. J. Mol. Sci..

[12] Cornelison R., Llaneza D.C., Landen C.N. (2017). Emerging Therapeutics to Overcome Chemoresistance in Epithelial Ovarian Cancer: A Mini-Review. Int. J. Mol. Sci..

[13] Alberts B., Johnson A., Lewis J., Raff M., Roberts K., Walter P. (2002). Molecular Biology of the Cell.

[14] Tapia G., Diaz-Padilla I., Diaz-Padilla I. (2013). Molecular mechanisms of platinum resistance in ovarian cancer. Ovarian Cancer—A Clinical and Translational Update.

[15] Lai Y.H., Kuo C., Kuo M.T., Chen H.H.W. (2018). Modulating chemosensitivity of tumors to platinum-based antitumor drugs by transcriptional regulation of copper homeostasis. Int. J. Mol. Sci..

[16] Cerovska E., Elsnerova K., Vaclavikova R., Soucek P. (2017). The role of membrane transporters in ovarian cancer chemoresistance and prognosis. Expert Opin. Drug Metab. Toxicol..

[17] Samimi G., Varki N.M., Wilczynski S., Safaei R., Alberts D.S., Howell S.B. (2003). Increase in expression of the copper transporter ATP7A during platinum drug-based treatment is associated with poor survival in ovarian cancer patients. Clin. Cancer Res..

[18] Nakayama K., Kanzaki A., Terada K., Mutoh M., Ogawa K., Sugiyama T., Takenoshita S., Itoh K., Yaegashi N., Miyazaki K. (2004). Prognostic value of the Cu-transporting ATPase in ovarian carcinoma patients receiving cisplatin-based chemotherapy. Clin. Cancer Res..

[19] Elsnerova K., Mohelnikova-Duchonova B., Cerovska E., Ehrlichova M., Gut I., Rob L., Bartunkova J., Bouda J., Dvorak P., Soucek P. (2016). Gene expression of membrane transporters: Importance for prognosis and progression of ovarian carcinoma. Oncol. Rep..

[20] Lee C.M., Lo H.W., Shao R.P., Wang S.C., Xia W., Gershenson D.M., Hung M.C. (2004). Selective activation of ceruloplasmin promoter in ovarian tumors: Potential use for gene therapy. Cancer Res..

[21] Chen J., Jiang Y., Shi H., Peng Y., Fan X., Li C. (2020). The molecular mechanisms of copper metabolism and its roles in human diseases. Pflugers Arch..

[22] Huang H., Li Y., Liu J., Zheng M., Feng Y., Hu K., Huang Y., Huang Q. (2012). Screening and Identification of Biomarkers in Ascites Related to Intrinsic Chemoresistance of Serous Epithelial Ovarian Cancers. PLoS ONE.

[23] Hojnik M., Šuster N.K., Smrkolj Š., Sisinger D., Grazio S.F., Verdenik I., Rižner T.L. (2022). AKR1B1 as a Prognostic Biomarker of High-Grade Serous Ovarian Cancer. Cancers.

[24] Mansoori B., Mohammadi A., Davudian S., Shirjang S., Baradaran B. (2017). The Different Mechanisms of Cancer Drug Resistance: A Brief Review. Adv. Pharm. Bull..

[25] Bell C.C., Gilan O. (2019). Principles and mechanisms of non-genetic resistance in cancer. Br. J. Cancer.

[26] Galluzzi L., Vitale I., Michels J., Brenner C., Szabadkai G., Harel-Bellan A., Kroemer G. (2014). Systems biology of cisplatin resistance: Past, present and future. Cell Death Dis..

[27] Katano K., Kondo A., Safaei R., Holzer A., Samimi G., Mishima M., Kuo Y.M., Rochdi M., Howell S.B. (2002). Acquisition of resistance to cisplatin is accompanied by changes in the cellular pharmacology of copper. Cancer Res..

[28] Kalayda G.V., Wagner C.H., Buss I., Reedijk J., Jaehde U. (2008). Altered localisation of the copper efflux transporters ATP7A and ATP7B associated with cisplatin resistance in human ovarian carcinoma cells. BMC Cancer.

[29] Samimi G., Safaei R., Katano K., Holzer A.K., Rochdi M., Tomioka M., Goodman M., Howell S.B. (2004). Increased expression of the copper efflux transporter ATP7A mediates resistance to cisplatin, carboplatin, and oxaliplatin in ovarian cancer cells. Clin. Cancer Res..

[30] De Feyter S., Beyens A., Callewaert B. (2023). ATP7A-related copper transport disorders: A systematic review and definition of the clinical subtypes. J. Inherit. Metab. Dis..

[31] Liu X., Zhong S., Yan L., Zhao H., Wang Y., Hu Y., Jia Y. (2020). Correlations Among mRNA Expression Levels of ATP7A, Serum Ceruloplasmin Levels, and Neuronal Metabolism in Unmedicated Major Depressive Disorder. Int. J. Neuropsychopharmacol..

[32] Kobal B., Noventa M., Cvjeticanin B., Barbic M., Meglic L., Herzog M., Bordi G., Vitagliano A., Saccardi C., Skof E. (2018). Primary debulking surgery versus primary neoadjuvant chemotherapy for high grade advanced stage ovarian cancer: Comparison of survivals. Radiol. Oncol..

[33] Škof E., Merlo S., Pilko G., Kobal B. (2016). The role of neoadjuvant chemotherapy in patients with advanced (stage IIIC) epithelial ovarian cancer. Radiol. Oncol..

[34] You B., Robelin P., Tod M., Louvet C., Lotz J.-P., Abadie-Lacourtoisie S., Fabbro M., Desauw C., Bonichon-Lamichhane N., Kurtz J.-E. (2020). CA-125 ELIMination rate constant K (KELIM) is a marker of Chemosensitivity in patients with ovarian Cancer: Results from the phase II CHIVA trial. Clin. Cancer Res..

[35] You B., Freyer G., Gonzalez-Martin A., Lheureux S., McNeish I., Penson R.T., Pignata S., Pujade-Lauraine E. (2021). The role of the tumor primary chemosensitivity relative to the success of the medical-surgical management in patients with advanced ovarian carcinomas. Cancer Treat. Rev..

[36] Rose P.G. (2022). Ovarian cancer recurrence: Is the definition of platinum sensitivity modified by PARPi, bevacizumab or other intervening treatments? A clinical perspective. Cancer Drug Resist..

[37] Chen F., Han B., Meng Y., Han Y., Liu B., Zhang B., Chang Y., Cao P., Fan Y., Tan K. (2021). Ceruloplasmin correlates with immune infiltration and serves as a prognostic biomarker in breast cancer. Aging.

[38] Lane D., Matte I., Garde-Granger P., Laplante C., Carignan A., Rancourt C., Piché A. (2015). Inflammation-regulating factors in ascites as predictive biomarkers of drug resistance and progression-free survival in serous epithelial ovarian cancers. BMC Cancer.

[39] Wells R.G., Feldman A.M., Groszmann R.J., Sass D.A. (2017). Pathophysiology of ascites. Ascites: Pathophysiology, Diagnosis, and Management.

[40] Nayak S.B., Bhat V.R., Upadhyay D., Udupa S.L. (2003). Copper and ceruloplasmin status in serum of prostate and colon cancer patients. Indian J. Physiol. Pharmacol..

[41] Patil M.B., Lavanya T., Kumari C.M., Shetty S.R., Gufran K., Viswanath V., Swarnalatha C., Babu J.S., Nayyar A.S. (2021). Serum ceruloplasmin as cancer marker in oral pre-cancers and cancers. J. Carcinog..

[42] Kluger H.M., Kluger Y., Gilmore-Hebert M., DiVito K., Chang J.T., Rodov S., Yang C.R., DeFatta R.J., Perry R.R., Geradts J. (2004). cDNA microarray analysis of invasive and tumorigenic phenotypes in a breast cancer model. Lab. Investig..

[43] Chakravarty P.K., Ghosh A., Chowdhury J.R. (1986). Evaluation of ceruloplasmin concentration in prognosis of human cancer. Acta Med. Okayama.

[44] Onizuka K., Migita S., Yamada H., Matsumoto I. (1999). Serum protein fractions in patients with laryngeal cancer undergoing radiation therapy: Possibility as a prognostic factor. Fukuoka Igaku Zasshi.

[45] Weijl N.I., Hopman G.D., Wipkink-Bakker A., Lentjes E.G., Berger H.M., Cleton F.J., Osanto S. (1998). Cisplatin combination chemotherapy induces a fall in plasma antioxidants of cancer patients. Ann. Oncol..

[46] Korenkova V., Slyskova J., Novosadova V., Pizzamiglio S., Langerova L., Bjorkman J., Vycital O., Liska V., Levy M., Veskrna K. (2016). The focus on sample quality: Influence of colon tissue collection on reliability of qPCR data. Sci. Rep..

[47] Blagden S.P. (2015). Harnessing Pandemonium: The Clinical Implications of Tumor Heterogeneity in Ovarian Cancer. Front. Oncol..

[48] Turajlic S., Swanton C. (2016). Metastasis as an evolutionary process. Science.

[49] Roberts C.M., Cardenas C., Tedja R. (2019). The Role of Intra-Tumoral Heterogeneity and Its Clinical Relevance in Epithelial Ovarian Cancer Recurrence and Metastasis. Cancers.

[50] Al-Bahlani S., Fraser M., Wong A.Y., Sayan B.S., Bergeron R., Melino G., Tsang B.K. (2011). P73 regulates cisplatin-induced apoptosis in ovarian cancer cells via a calcium/calpain-dependent mechanism. Oncogene.

